# Perceptions and attitudes of bhutanese people on *Sowa Rigpa*, traditional bhutanese medicine: a preliminary study from Thimphu

**DOI:** 10.1186/1746-4269-7-3

**Published:** 2011-01-10

**Authors:** Namgay Lhamo, Sabine Nebel

**Affiliations:** 1 National Institute of Traditional Medicine, Royal University of Bhutan, Thimphu, Bhutan; 2 Alte Landstrasse, Rueschlikon, Switzerland

## Abstract

**Background:**

Many claims are made that the use of traditional medicine is a substantial and growing part of healthcare behavior around the world. In Bhutan traditional medical practice is one of the country's tangible heritages. The country hosts two forms of traditional medicines: local healing practices and the official traditional medical system known as *sowa rigpa*, meaning "the science of healing". This paper explores the attitudes on *sowa rigpa *among Bhutanese living in Thimphu, the capital of Bhutan.

**Methods:**

This study was conducted from May to September 2009. In total, 155 people coming from diverse social backgrounds were randomly selected for the study. The study made use of qualitative as well as quantitative approaches, involving the administration of questionnaires and conducting in-depth interviews.

**Results:**

From the 155 respondents 99% have heard about *sowa rigpa*, mainly from their friends or relatives. The study showed that *sowa rigpa *is popular among the respondents since more than half (51%) have said that they have been treated by *sowa rigpa *doctors. The data revealed that the majority (83%) of the respondents are satisfied with the treatment received.

**Conclusion:**

The Bhutanese healthcare system that integrates *sowa rigpa *and modern medicine offers an opportunity for active healthcare decision-making by the patients. The improved understanding of the knowledge, attitudes and treatment seeking practices of the participants in the study provides useful information for health practitioners and policy makers to plan health activities. The present preliminary study represents only people living in the capital city of Bhutan. Therefore, a further nationwide study is planned to better understand the role *sowa rigpa *plays also in rural Bhutan.

## Background

Despite the advances in modern medicine, many people around the world seek traditional medicine to cure various health problems. Because of its recent upsurge in popularity in the West, traditional medicine looks set to become "a permanent feature of the cultural landscape" (p.49) [1]. Much social science research has focused on the growing demand and use of traditional medicine [1-4]. These studies have variously attributed this upsurge to the dissatisfaction among patients with modern medicine, a desire for holistic treatment that value patient experience, or the emergence of the "smart consumers" seeking empowerment through active healthcare decision-making. There have been many claims that the use of traditional medicine is a substantial and growing part of healthcare behavior [5]. Kuhn [6] and Bright [7] claim that in addition to modern biomedicine, traditional medicine provides healthcare to 65-85% of the world's population in developing as well as developed nations. A recent report of the World Health Organization [8] indicates that, for example 75% of the French, 30% of the Vietnamese and 40% of the Indonesian population use traditional medicine.

Bhutan, situated in the Eastern Himalayas, between China in the north and India to the south, is a landlocked country characterized by high mountains and a rugged terrain. Bhutan has an estimated population of approximately 700'000 people [9], of which 70% live in rural areas. The country hosts two forms of traditional medicine: local healing practices and the official traditional medical system known as *sowa rigpa*. *Sowa rigpa *is recognized as one of the country's tangible heritage [10]. This traditional medical system is one of the five major sciences of Tibetan Buddhism (*rigs gnas che ba Inga*) and blends philosophy, culture and Buddhism. Thus, in Bhutanese thinking that is heavily influenced by Buddhism, health and spirituality are seen as inseparable aspects and together they reveal the true origin of any illness. This art of healing is therefore a holistic approach to healthcare [11]. *Sowa rigpa *in Bhutan is based on principles of Indian and Tibetan Medicine and has also incorporated some ancient local medical practices [12-14]. According to Kleinman, a healthcare system articulates illness as a cultural idiom, linking perceptions about disease causation, the experience of symptoms, decisions concerning treatment alternatives, and actual therapeutic practices [15].

Today, the healthcare system in Bhutan comprises modern and traditional medical systems, which are integrated to maximize public healthcare services. The *sowa rigpa *system was formally introduced into the national healthcare system in 1967 under the command of the third King Jigme Dorji Wangchuck. This was initiated with the vision of not only looking after the medical welfare of people but also of preserving and further promoting the traditional medical system in the country [16]. Starting from a single indigenous dispensary in 1967 in the capital city Thimphu, the traditional medical service in Bhutan has grown rapidly over the years to cover the entire country. In 1979 the indigenous dispensary was upgraded to the National Indigenous Hospital and in 1998 to the Institute of Traditional Medicine Services (ITMS) with respect to the increased interest in *sowa rigpa*. The ITMS has three functional units: the National Traditional Medicine Hospital responsible for the development and provision of traditional medical care; the National Institute of Traditional Medicine responsible for the development of human resources required to provide traditional medicine services; and the Pharmaceutical Research Unit responsible for the manufacturing and production of traditional medicines [17].

By the end of 2001, traditional medicine units (providing *sowa rigpa*) were attached to district hospitals, and had been established in all 20 districts of Bhutan. Thus the collaboration between modern and traditional medicine and cross referrals of patients was facilitated. The district traditional medicine unit is staffed by one *drungtsho *(traditional physician) and one *menpa *(clinical assistant). According to Wangchuk [18], 20-30% of the daily outpatients are being treated with *sowa rigpa*. The National Traditional Medicine Hospital in Thimphu is not only responsible for tertiary healthcare services but also serves as a referral center for all the district traditional medicine units in the country. The institute offers a wide range of treatments such as golden and silver needle therapy, blood letting, moxabustion, herbal bath, herbal steam application and bath, nasal lavage, and massage with medicated oil [16]. However none of these therapies are available at the district level, except for gold and silver needle therapy. The types of medicines administered are different herbal compounds in the form of pills, tablets, capsules, syrups, ointments, medicated oil, or powder [19]. The traditional medical system of *sowa rigpa *contains more than one thousand herbal formularies and recipes [20].

As *sowa rigpa *considers the health of the entire person, it is generally believed to be particularly effective in curing chronic diseases such as sinusitis, arthritis, asthma, rheumatism, liver problems and diseases related to the digestive and nervous systems [18]. About 30,000 patients are treated annually, which is a significant proportion of the total number of patients in the country [19]. Although the increasing number of patients visiting the *sowa rigpa *centers serves as a good indicator, no empirical study has yet been carried out to analyze the role of *sowa rigpa *in Bhutanese society. Although *sowa rigpa *has been catering to the health needs of its patients from time immemorial, not much is really known about its contribution to the healthcare system of the society. Often, people tend to attribute their well-being either to the more prominent modern medicine or to other forms of local healings, such as religious or spiritual practices. This preliminary study explores the perceptions and attitudes of Bhutanese people on *sowa rigpa*, to better understand the awareness, treatment seeking practices, common illnesses treated, and the level of trust and satisfaction of people with this system of medicine.

## Methods

The study was conducted from May to September 2009 by the authors and was supported by the National Institute of Traditional Medicine in Thimphu, Bhutan.

### Data collection

The study was designed to make use of both qualitative as well as quantitative approaches, which is specifically termed as the concurrent triangulation strategy of the mixed method (p. 217) [21]. It involved combining open-ended questions with closed-ended questions on the survey. The researchers chose this model using two complementary methods in an attempt to confirm, cross-validate, or corroborate findings within a single study [22].

To collect the data, 200 people living in Thimphu with diverse background in terms of their level of education, age, sex, marital status, occupation and location of their parent districts were randomly selected and asked whether they would like to participate in this study. A simple random sampling was done using the telephone directory of the city. Questionnaires, written in English, were administered to all the participants who could read and write English. Prior to the actual administration of the questionnaires, the questionnaire was tested with seven people. This was done to determine the strengths or weaknesses of the survey concerning question format, wording and order. For participants illiterate in English face-to-face interviews were conducted by the researchers in Dzongkha, the national language of Bhutan. The same questions as in the questionnaire were asked, adhering strictly to the interview guidelines of the study.

In addition, to gain a deeper and more detailed understanding about people's attitude on *sowa rigpa*, semi-structured in-depth interviews were conducted with five of the participants, who had a deeper understanding of *sowa rigpa*. Key areas of these interviews included the participants' views, their experience, their definition of *sowa rigpa*, and the use of *sowa rigpa *services in their families and communities.

Informed oral consent was sought from the participants prior to the administration of the questionnaire. The study was anonymous and voluntary, and informants were free to withdraw at any time. All the information was recorded verbatim and subsequently transcribed.

### Data analysis

Collected data were coded and entered into SPSS 13.0. Responses to the questionnaire were analyzed descriptively by sex, age, and level of education. For the qualitative questions, data were analyzed using phenomenographic techniques described by Dahlgren and Fallsberg 1991 [23].

## Results and Discussion

From the 200 people invited to participate in the study, 155 people had responded (response rate 78%). The majority of the data were collected through questionnaires in English, and 12 interviews were conducted in Dzongkha. In addition, five respondents who had a deeper understanding of *sowa rigpa *were asked to participate in semi-structured in-depth interviews.

Of the 155 respondents, 44% were females (n = 68), 55% were males (n = 85) and two missing data. Participants' age ranged from 18-88 years. Of the 155 respondents, 18% were below the age of 25, a majority (over 61%) of the respondents fell in the age group of 25-54 years, 10% were between 55-64 years and 11% of the respondents were 65 years and above. The education level of the participants ranged from uneducated (16%), i.e. people who have never been to school, to people who had attended higher education (55%) such as bachelors, masters or PhD degrees.

### Informants' knowledge about sowa rigpa

When asked about their knowledge of *sowa rigpa*, 99% of the respondents said that they had heard about *sowa rigpa*. They gave short descriptions of what they understood about *sowa rigpa*. The study further attempted to find out how people first came to know about *sowa rigpa*. When enquiring about the sources of information, a majority of the respondents said that they have heard about *sowa rigpa *from their friends (43%). This was followed by relatives (38%), *sowa rigpa *centers (28%), media (27%), self-study (24%) and seniors (19%). This indicates that *sowa rigpa *in Bhutan is an important topic in society as people are discussing about it among friends, relatives and people at the workplace. Moreover, the findings indicate that media play an important role in creating awareness among the Bhutanese people about *sowa rigpa *and its aspects.

### Treatment seeking practices of Bhutanese living in Thimphu

The study attempted to find out about the treatment seeking practices of Bhutanese living in Thimphu. To address the issue, attention was focused on four questions: 1) Have you ever treated an illness with *sowa rigpa*? 2) Have you or any of your family members visited a *sowa rigpa *center during the past one year? 3) Have you ever experienced any side-effects of from *sowa rigpa *medicines? 4) Why do you choose to visit *sowa rigpa *clinic instead of a modern medical center?

**Figure 1:**
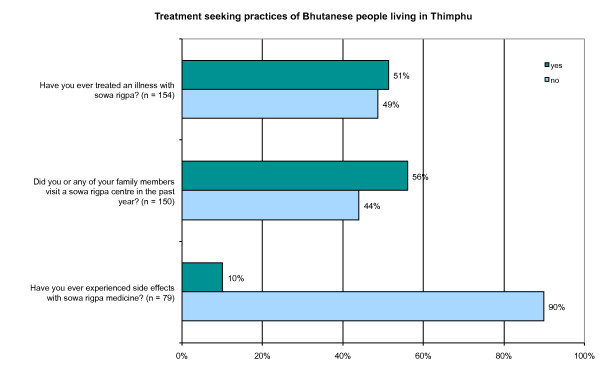
**Treatment seeking practices of Bhutanese people living in Thimphu**.

**Figure 2:**
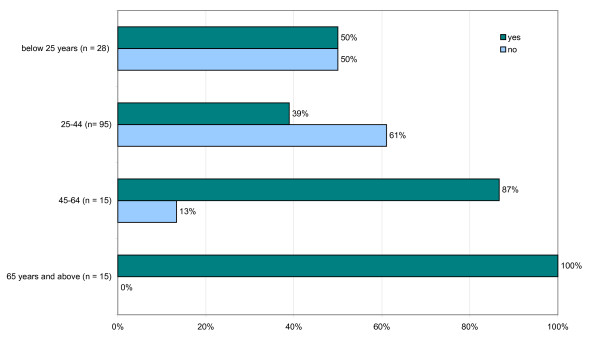
**Age distribution of respondents having taken *sowa rigpa *to treat an illness (n = 153)**.

The findings of the study indicate that *sowa rigpa *is quite popular among Thimphu residents since half (51%) the respondents said that they have taken treatment from *sowa rigpa *(Figure 1). Besides, it is very interesting to find out that treatment by *sowa rigpa *is sought by people of all ages, young and old (Figure 2). Hence the findings of the present study proves the common notion wrong that "only old and aging people seek treatment from *sowa rigpa"*. Furthermore, the data showed that 83% of the uneducated participants (n = 30) used *sowa rigpa*, while only 40% of the participants with higher education (n = 84) and 50% of the participants who attended school up to class XII (n = 38) said to have ever used *sowa rigpa*.

Respondents stated various reasons for their decision to visit a *sowa rigpa *center instead of a modern medical centre which centred around the following themes:

a) Sowa rigpa medicines are herbal and have no side effects;

b) Sowa rigpa is effective for the treatment of many illnesses;

c) Sowa rigpa is sought as an alternative treatment;

d) Sowa rigpa is traditional and based on Buddhism;

*e) Friends/relatives said that sowa rigpa was very effective*.

The above key responses suggest that one of the main factors supporting the popularity of *sowa rigpa *is the general perception that "herbal means safe". While the question of whether *sowa rigpa *has side-effects remains unexplored, the present study shows that almost all respondents (91%) who had taken treatment with *sowa rigpa *did not experience any side-effects (Figure 1). Moreover, the very few respondents (9%) who had experienced some side-effects said that the effects were mild, such as mild headache, or giddiness, and that they were not too sure whether to attribute those problems to *sowa rigpa*.

Although *sowa rigpa *has been used for ages, there is a lack of clinical evidence to prove its efficacy and safety. Quality control and safety of herbal medicine remains expensive and complex, in particular it is difficult for *sowa rigpa *since compounded medicines are involved. Also the concept that "natural means safe" often leads to misuse of traditional medicine by the public. Thus, clinical and pre-clinical research needs to be carried out in the future, to address the question of efficacy and safety of *sowa rigpa*.

### Diseases treated by sowa rigpa

**Figure 3:**
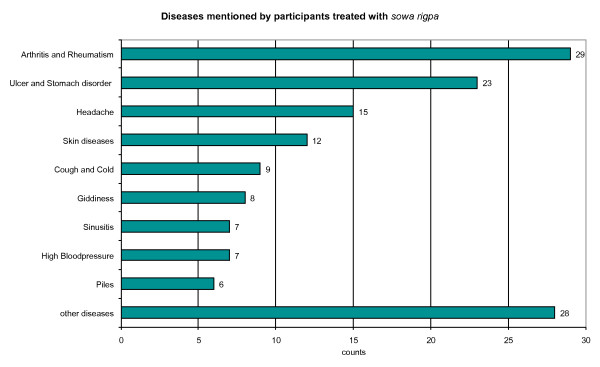
**Diseases mentioned by participants treated with *sowa rigpa***.

As *sowa rigpa *diagnoses grow out of a different rational and cannot be easily translated into biomedical terminology, reference to the handbook "Bhutanese Traditional Classification of Diseases & Related Health Problems" [24] was made to classify the illnesses reported by the participants. The data reveal that *sowa rigpa *is popular among the patients for the treatment of diseases such as arthritis and rheumatism, ulcer and stomach disorder, headache, and skin diseases (Figure 3). This result is reflected in parts in the list of the top ten diseases recorded by the Institute of Traditional Medicine Hospital (ITMH) in Thimphu for the year 2008. The top ten diseases listed are neurological problems, sinusitis, ulcer and stomach disorders, gastric problems, arthritis and rheumatism, skin diseases, blood pressure, cough and cold, chronic injuries and combination of gastric problems and pressure.

### People's attitude on sowa rigpa

The term "attitude" in this paper is used to describe people's opinion about *sowa rigpa *and therefore we use the terms "satisfaction" and "trust", to study people's attitude on *sowa rigpa*.

**Figure 4:**
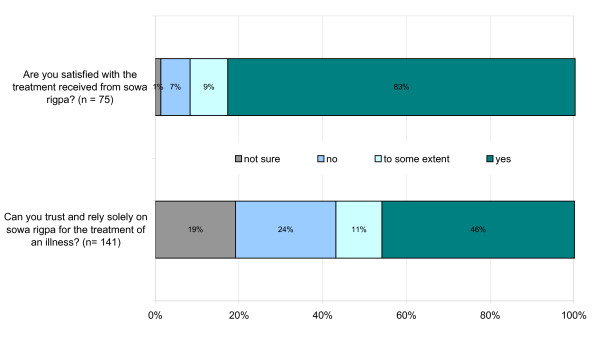
**Respondents' level of satisfaction regarding *sowa rigpa *(n = 75) and level of trust in *sowa rigpa *(n = 141)**.

When asked whether they were satisfied with the treatment received from *sowa rigpa*, a majority of informants (83%) said yes that they were satisfied, 10% were only satisfied to some extend or were not sure, while only 7% of the respondents said that they were not satisfied (Figure 4). This data show that people benefit from the healthcare services offered by *sowa rigpa*. This is further supported by the following statement made by a participant in the in-depth interviews: "*I am a fan of sowa rigpa. I have benefited a lot from it. I don't trust modern medicine since it uses a lot of chemicals to suppress the pain. Sowa rigpa helps to cure the illness gradually and permanently without any side-effects*."

While the level of satisfaction with the services received from *sowa rigpa *is encouraging, one of the findings indicates that a significant number of people (43%) are skeptical or not sure about whether they can trust and solely rely on *sowa rigpa *for their health-care. Moreover, when trying to find out the health-seeking pattern, a majority of the respondents (61%) said that they used other drugs besides *sowa rigpa*. Some of the most commonly used drugs were pain killers, such as paracetamol, ibuprofen, or aspirin, antibiotics, cough mixtures and histamine H_2_-receptor antagonists. Further, when asked whether *sowa rigpa *can substitute modern medicine, the majority of the respondents (58%) said "no". One may find it contradictory to say that people are satisfied with the treatment received from *sowa rigpa *but they are not able to trust and fully rely on it. However, these seemingly contradictory findings can be explained by the following reasons that informants stated for not being able to trust and rely solely on *sowa rigpa*.

The theme reasons were:

a) Sowa rigpa cannot cure all the diseases but only certain ones;

b) Lack of adequate facilities for sowa rigpa;

c) Modern medicines are better equipped for emergencies and surgeries;

d) Treatment is time consuming with sowa rigpa;

e) Many people are highly dependent on modern medicine;

d) Diagnosis of diseases is better with modern medicine;

*e) Sowa rigpa is not research based (i.e. not scientifically proven to be effective)*.

The present study was also interested in finding out the scope of *sowa rigpa *in the country for the years to come. Of 146 respondents, only 1% said that *sowa rigpa *services should be stopped; while 99% strongly stated that it should not only be continued but also further developed and improved.

The views and opinions expressed by the participants in the in-depth interviews suggest that *sowa rigpa *plays a vital role in the healthcare of the country. Although it takes a long time and thus a lot of patience to cure diseases people have developed faith in it. They had many success stories to share about *sowa rigpa*. For instance, there was one participant who remarked, *"I would have left this world long time back, had it not been for our sowa rigpa"*. Data indicate that although *sowa rigpa *is used as an ultimate treatment people still have high regard for it, as evidenced by the following remark by one of the participants: "*As I did not get cured by modern medicine, I started taking traditional medicine and I feel that it has helped me a lot*".

From the in-depth interviews researchers have learned that *sowa rigpa *and local healing practices were the only means of healthcare available to the Bhutanese in the past. When modern medicine was gradually introduced in Bhutan in the 1960 s, the popularity of *sowa rigpa *went down, as people got a choice between the two systems. An elderly informant remarked, "*we had no alternatives in the past, as sowa rigpa and local practices were the only remedy for the treatment of any health problems. But I still seek treatment form sowa rigpa today, because it is strongly rooted in Buddhism. The medicines are blessed by the medicine Buddha."*

Many people have faith in traditional medicine and seek treatment from it. The inclusion of *sowa rigpa *in the healthcare system provides alternative choices to the patients. In Bhutan, *sowa rigpa *is also widely regarded as a symbol of cultural heritage that needs preservation and further promotion.

## Conclusion

The findings of the present study showed that the knowledge and awareness on *sowa rigpa *by Bhutanese people living in Thimphu is good. It has been found that a significant number of respondents seek treatment from *sowa rigpa*. Results indicated that treatment is sought by all ages, young and old and also across different levels of education. Therefore, *sowa rigpa *is shown to be popular not only among the aging population of the country but also among the younger ones. The integration of *sowa rigpa *with the modern healthcare system not only adds dimensions to the nation's system of healthcare but also facilitates empowerment of patients by providing them with a choice of healthcare systems and different options for treatments.

The study also showed that participants' attitudes and perceptions on *sowa rigpa *are generally positive. However, people also expressed that they cannot solely rely on it as it lacks adequate facilities in terms of emergencies and surgical operations. People are of the opinion that *sowa rigpa *clinics be further expanded and better equipped so that Bhutanese people can enjoy better healthcare services of both traditional medicine and modern medicine. Since the present study focused only on people living in the capital city of Bhutan, a further nationwide study is necessary to better understand the kind of role *sowa rigpa *plays in the arena of Bhutan's public healthcare system.

Given the freedom for patients to make their own medical choice, empirical studies such as this have become imperative to gain a better understanding of patients' knowledge and attitudes and of treatment seeking practices. The findings of this and further studies are expected to be useful for health practitioners and policy makers to design and plan health policies and programs that meet the needs of patients. This will contribute to improving healthcare by building culturally sensitive, exemplary healthcare services for the benefit of the Bhutanese society.

## Competing interests

The authors declare that they have no competing interests.

## Consent

Written informed consent was obtained for publication of the accompanying images. A copy of the written consent is available for review by the Editor-in-Chief of this journal.

## Authors' contributions

Author NL designed the study, drafted and finalized the manuscript with SN. The author SN supported NL during the research project, drafted and finalized the manuscript with NL. Both authors read and approved the final manuscript.
